# Supplementary material for: Unveiling the Bioactive Potential of the Invasive Jellyfish Phyllorhiza punctata Through Integrative Transcriptomic and Proteomic Analyses
Source: Biomolecules. 2025 Aug 4;15(8):1121. doi: 10.3390/biom15081121 (PMC12383608; doi:10.3390/biom15081121)
Supplement: Supplementary file 1 [file biomolecules-15-01121-s001.zip › Suplementary Material/FileS3/PEPFOLD4/XP_065060389_1621/clusters.html]

 PEP-FOLD clusters


Download archive of all models  here   

|  |  |  |
| --- | --- | --- |
| # | file | sOPEP |
| # cluster 1 - Size: 9 |  |  |
|  | XP\_065060389\_1621-model1 | -5.83116 |
|  | XP\_065060389\_1621-model1.1 | -5.42632 |
|  | XP\_065060389\_1621-model1.2 | -5.05347 |
|  | XP\_065060389\_1621-model1.3 | -5.01762 |
|  | XP\_065060389\_1621-model1.4 | -4.98853 |
|  | XP\_065060389\_1621-model1.5 | -4.95224 |
|  | XP\_065060389\_1621-model1.6 | -4.62307 |
|  | XP\_065060389\_1621-model1.7 | -4.09787 |
|  | XP\_065060389\_1621-model1.8 | -3.9401 |
| # cluster 2 - Size: 5 |  |  |
|  | XP\_065060389\_1621-model2 | -5.50777 |
|  | XP\_065060389\_1621-model2.1 | -5.39072 |
|  | XP\_065060389\_1621-model2.2 | -5.33918 |
|  | XP\_065060389\_1621-model2.3 | -4.12188 |
|  | XP\_065060389\_1621-model2.4 | -4.0111 |
| # cluster 3 - Size: 2 |  |  |
|  | XP\_065060389\_1621-model3 | -5.49713 |
|  | XP\_065060389\_1621-model3.1 | -4.43444 |
| # cluster 4 - Size: 3 |  |  |
|  | XP\_065060389\_1621-model4 | -4.7623 |
|  | XP\_065060389\_1621-model4.1 | -4.6294 |
|  | XP\_065060389\_1621-model4.2 | -4.51459 |
| # cluster 5 - Size: 28 |  |  |
|  | XP\_065060389\_1621-model5 | -4.606 |
|  | XP\_065060389\_1621-model5.1 | -4.50882 |
|  | XP\_065060389\_1621-model5.2 | -4.42498 |
|  | XP\_065060389\_1621-model5.3 | -4.36393 |
|  | XP\_065060389\_1621-model5.4 | -4.31619 |
|  | XP\_065060389\_1621-model5.5 | -4.29685 |
|  | XP\_065060389\_1621-model5.6 | -4.25121 |
|  | XP\_065060389\_1621-model5.7 | -4.24721 |
|  | XP\_065060389\_1621-model5.8 | -4.21002 |
|  | XP\_065060389\_1621-model5.9 | -4.20979 |
|  | XP\_065060389\_1621-model5.10 | -4.13346 |
|  | XP\_065060389\_1621-model5.11 | -4.12981 |
|  | XP\_065060389\_1621-model5.12 | -4.07548 |
|  | XP\_065060389\_1621-model5.13 | -4.05673 |
|  | XP\_065060389\_1621-model5.14 | -4.0475 |
|  | XP\_065060389\_1621-model5.15 | -4.04554 |
|  | XP\_065060389\_1621-model5.16 | -4.03646 |
|  | XP\_065060389\_1621-model5.17 | -4.00616 |
|  | XP\_065060389\_1621-model5.18 | -4.00372 |
|  | XP\_065060389\_1621-model5.19 | -3.99361 |
|  | XP\_065060389\_1621-model5.20 | -3.99222 |
|  | XP\_065060389\_1621-model5.21 | -3.96771 |
|  | XP\_065060389\_1621-model5.22 | -3.94597 |
|  | XP\_065060389\_1621-model5.23 | -3.90004 |
|  | XP\_065060389\_1621-model5.24 | -3.89736 |
|  | XP\_065060389\_1621-model5.25 | -3.85679 |
|  | XP\_065060389\_1621-model5.26 | -3.84544 |
|  | XP\_065060389\_1621-model5.27 | -3.68871 |
| # cluster 6 - Size: 1 |  |  |
|  | XP\_065060389\_1621-model6 | -4.48639 |
| # cluster 7 - Size: 2 |  |  |
|  | XP\_065060389\_1621-model7 | -4.46007 |
|  | XP\_065060389\_1621-model7.1 | -4.07475 |
| # cluster 8 - Size: 32 |  |  |
|  | XP\_065060389\_1621-model8 | -4.31254 |
|  | XP\_065060389\_1621-model8.1 | -4.23343 |
|  | XP\_065060389\_1621-model8.2 | -4.224 |
|  | XP\_065060389\_1621-model8.3 | -4.19302 |
|  | XP\_065060389\_1621-model8.4 | -4.18486 |
|  | XP\_065060389\_1621-model8.5 | -4.17007 |
|  | XP\_065060389\_1621-model8.6 | -4.14035 |
|  | XP\_065060389\_1621-model8.7 | -4.1042 |
|  | XP\_065060389\_1621-model8.8 | -4.09734 |
|  | XP\_065060389\_1621-model8.9 | -4.09161 |
|  | XP\_065060389\_1621-model8.10 | -4.09161 |
|  | XP\_065060389\_1621-model8.11 | -4.04084 |
|  | XP\_065060389\_1621-model8.12 | -4.04084 |
|  | XP\_065060389\_1621-model8.13 | -4.02354 |
|  | XP\_065060389\_1621-model8.14 | -3.98543 |
|  | XP\_065060389\_1621-model8.15 | -3.98375 |
|  | XP\_065060389\_1621-model8.16 | -3.98017 |
|  | XP\_065060389\_1621-model8.17 | -3.97273 |
|  | XP\_065060389\_1621-model8.18 | -3.96052 |
|  | XP\_065060389\_1621-model8.19 | -3.91784 |
|  | XP\_065060389\_1621-model8.20 | -3.8761 |
|  | XP\_065060389\_1621-model8.21 | -3.87428 |
|  | XP\_065060389\_1621-model8.22 | -3.86865 |
|  | XP\_065060389\_1621-model8.23 | -3.82413 |
|  | XP\_065060389\_1621-model8.24 | -3.79694 |
|  | XP\_065060389\_1621-model8.25 | -3.78752 |
|  | XP\_065060389\_1621-model8.26 | -3.77464 |
|  | XP\_065060389\_1621-model8.27 | -3.77454 |
|  | XP\_065060389\_1621-model8.28 | -3.77331 |
|  | XP\_065060389\_1621-model8.29 | -3.77179 |
|  | XP\_065060389\_1621-model8.30 | -3.64773 |
|  | XP\_065060389\_1621-model8.31 | -3.62566 |
| # cluster 9 - Size: 8 |  |  |
|  | XP\_065060389\_1621-model9 | -4.23303 |
|  | XP\_065060389\_1621-model9.1 | -4.22636 |
|  | XP\_065060389\_1621-model9.2 | -4.15041 |
|  | XP\_065060389\_1621-model9.3 | -3.92208 |
|  | XP\_065060389\_1621-model9.4 | -3.89053 |
|  | XP\_065060389\_1621-model9.5 | -3.84955 |
|  | XP\_065060389\_1621-model9.6 | -3.82461 |
|  | XP\_065060389\_1621-model9.7 | -3.79355 |
| # cluster 10 - Size: 2 |  |  |
|  | XP\_065060389\_1621-model10 | -4.10459 |
|  | XP\_065060389\_1621-model10.1 | -3.9901 |
| # cluster 11 - Size: 1 |  |  |
|  | XP\_065060389\_1621-model11 | -4.06071 |
| # cluster 12 - Size: 3 |  |  |
|  | XP\_065060389\_1621-model12 | -3.89358 |
|  | XP\_065060389\_1621-model12.1 | -3.81327 |
|  | XP\_065060389\_1621-model12.2 | -3.69617 |
| # cluster 13 - Size: 2 |  |  |
|  | XP\_065060389\_1621-model13 | -3.88471 |
|  | XP\_065060389\_1621-model13.1 | -3.72407 |
| # cluster 14 - Size: 1 |  |  |
|  | XP\_065060389\_1621-model14 | -3.68562 |
| # cluster 15 - Size: 1 |  |  |
|  | XP\_065060389\_1621-model15 | -3.66162 |
|  |  |  |
